# Clinical analysis of EV‐Fingerprint to predict grade group 3 and above prostate cancer and avoid prostate biopsy

**DOI:** 10.1002/cam4.6216

**Published:** 2023-06-17

**Authors:** Adrian Fairey, Robert J. Paproski, Desmond Pink, Deborah L. Sosnowski, Catalina Vasquez, Bryan Donnelly, Eric Hyndman, Armen Aprikian, Adam Kinnaird, Perrin H. Beatty, John D. Lewis

**Affiliations:** ^1^ Kipnes Urology Centre, Kaye Edmonton Clinic Edmonton Alberta Canada; ^2^ Nanostics Inc. Edmonton Alberta Canada; ^3^ Department of Oncology Katz Group Centre, University of Alberta Edmonton Alberta Canada; ^4^ Prostate Cancer Centre University of Calgary Calgary Alberta Canada; ^5^ Department of Surgery McGill University, Montreal General Hospital Montreal Quebec Canada

**Keywords:** biomarkers, clinical cancer research, prostate cancer, screening

## Abstract

**Background:**

There is an unmet clinical need for minimally invasive diagnostic tests to improve the detection of grade group (GG) ≥3 prostate cancer relative to prostate antigen‐specific risk calculators. We determined the accuracy of the blood‐based extracellular vesicle (EV) biomarker assay (EV Fingerprint test) at the point of a prostate biopsy decision to predict GG ≥3 from GG ≤2 and avoid unnecessary biopsies.

**Methods:**

This study analyzed 415 men referred to urology clinics and scheduled for a prostate biopsy, were recruited to the APCaRI 01 prospective cohort study. The EV machine learning analysis platform was used to generate predictive EV models from microflow data. Logistic regression was then used to analyze the combined EV models and patient clinical data and generate the patients' risk score for GG ≥3 prostate cancer.

**Results:**

The EV‐Fingerprint test was evaluated using the area under the curve (AUC) in discrimination of GG ≥3 from GG ≤2 and benign disease on initial biopsy. EV‐Fingerprint identified GG ≥3 cancer patients with high accuracy (0.81 AUC) at 95% sensitivity and 97% negative predictive value. Using a 7.85% probability cutoff, 95% of men with GG ≥3 would have been recommended a biopsy while avoiding 144 unnecessary biopsies (35%) and missing four GG ≥3 cancers (5%). Conversely, a 5% cutoff would have avoided 31 unnecessary biopsies (7%), missing no GG ≥3 cancers (0%).

**Conclusions:**

EV‐Fingerprint accurately predicted GG ≥3 prostate cancer and would have significantly reduced unnecessary prostate biopsies.

## Graphical abstract

We developed an accurate diagnostic blood test for grade group (GG) ≥3 prostate cancer that comprises the generation of a microflow cytometry dataset of three prostate cancer extracellular vesicle (EV) biomarkers, analysis with a novel machine learning algorithm to generate predictive EV models, and logistic regression analysis of these models with patient clinical data to calculate the risk score for the probability of GG ≥3 prostate cancer.

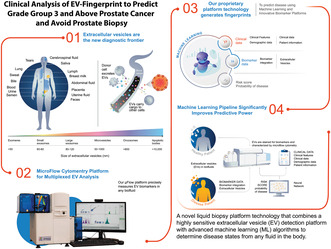

## 1 INTRODUCTION

Prostate cancer is the most diagnosed cancer in men and the second leading cause of male cancer deaths in the US.^1^, ^2^, ^3^ Most men are diagnosed with low‐grade, indolent, localized, or regional prostate cancer and have a 5‐year net survival rate of 100%. However, the 5‐year survival rate drops to 32% for men diagnosed with high‐grade, metastatic, clinically significant prostate cancer.^1^, ^2^ Prostate cancer cells are graded for defining cancerous features compared to normal prostate cells using the Gleason Score Grade Group (GG) system.^4^, ^5^, ^6^

The prostate‐specific antigen (PSA) blood test is the current prostate cancer screening tool, and at the standard cutoff has a high negative predictive value (85% NPV), but low specificity for GG ≥3 prostate cancer.^7^ Therefore, there is a critical need for a more specific prostate cancer biomarker test to counter the overdiagnosis of GG 1 and GG 2 prostate cancer identified from PSA testing.^8^, ^9^ Guidelines for early diagnosis of prostate cancer recommend using biomarker tests or multi‐parametric magnetic resonance imaging (mpMRI), in addition to PSA, to improve the sensitivity and specificity for screening for prostate cancer before biopsy.^10^, ^11^, ^12^, ^13^

Extracellular vesicles (EVs) are vesicles secreted by healthy and cancerous cells of various sizes (30–2000 nm), formation (endosome, plasma membrane, apoptosis),^14^ location (all tissues and biofluids), and description (exosomes, microvesicles, microparticles, apoptotic particles, apoptotic bodies, and oncosomes).^15^ EVs carry molecular cargo within biological fluids and function in cellular communication and maintenance, making them influential in determining cellular phenotype and disease progression.^15^ Clinically, EVs are a complex biological resource that can be probed for diagnostic and prognostic biomarkers to determine the patients' physiological disease‐state.^16^

We recently reported the development of an EV machine learning platform (EVMAP) that can be used to generate risk scores for cancers and other diseases.^17^ In this study we hypothesized that augmenting the predictive power of PSA with other clinically useful data, such as a risk score calculator or another liquid sample test, may be highly effective at increasing GG ≥3 prostate cancer sensitivity and specificity and reducing unnecessary biopsies. We used our EVMAP technology to create an accurate diagnostic blood test for GG ≥3 prostate cancer, called the EV‐Fingerprint test. The test comprises the generation of a microflow cytometry (μFCM) dataset of three prostate cancer EV‐biomarkers, dataset analysis with EVMAP to generate predictive EV models, and logistic regression analysis of these models with the PCPTRC 2.0 high grade cancer probability as the clinical data input to calculate the probability of GG ≥3 prostate cancer.^18^

## 2 MATERIALS AND METHODS

### 2.1 Patients

Patients were included from June 2014 to January 2017 in Alberta and followed for up to 5 years according to the Alberta Prostate Cancer Research Initiative (APCaRI) Standard Operating Procedures.^19^ The inclusion criteria were (a) adult male residents of Alberta, Canada (≥18 years) without prior prostate cancer diagnosis, referred to urology clinics in Alberta for prostate evaluation after high levels of blood PSA were detected and an abnormal digital rectal exam (DRE) performed by urologists, and scheduled for a prostate biopsy for diagnosis, (b) willing to permit provincial health agencies to disclose health‐related information to study, and (c) sign the informed consent form. The exclusion criteria were (a) unwilling to participate in the study, (b) unavailable for standard clinical urological care or follow‐up in Alberta, (c) prior diagnosis of prostate cancer, or (d) presentation of metastatic prostate cancer.

### 2.2 Ethics

All patients in the cohort gave written informed consent and the study was approved by the Health Research Ethics Board of Alberta Cancer Committee. The study methodologies conformed to the standards set by the Declaration of Helsinki.^20^ The EV‐Fingerprint test results were not provided to the clinical sites for patient care, and the laboratory personnel who performed the tests were blinded for patient characteristics.

### 2.3 Blood procurement

Blood procurement and sample preservation was done following the APCaRI standardized 2‐h arm to freezer protocol.^19^ Specimens included in this study were not subjected to freeze–thaw cycles.

### 2.4 Biopsy, radiology and pathology

Standard transrectal ultrasound guided, 12 core prostate biopsies were performed, and Gleason group grading was done following recommendations from the 2019 International Society of Urological Pathology (ISUP) Consensus Conference on Grading of Prostatic Carcinoma.^21^, ^22^

### 2.5 Antibody and probe labeling of EVs

Frozen plasma samples were thawed and centrifuged at 16,000 rcf for 30 min to remove large debris and platelet particles and generate platelet‐poor plasma. Two primary antibodies and one peptide probe, conjugated to different fluorescent labels, were used to label EVs in patient plasma supernatant samples using the following procedure. A 10‐μL aliquot of platelet‐poor plasma was incubated with 0.4 μg of mouse anti‐human prostate‐specific membrane antigen (PSMA) monoclonal antibody clone J591 (Cornell University) and 0.133 mM Cy5‐conjugated 18 amino acid ghrelin peptide (Luyt Lab, Western University) for 30 min, followed by a F(ab’)2‐Goat anti‐Mouse IgG (H + L) Secondary Antibody, Qdot® 565 conjugate for an additional 30 min. A separate reaction combined mouse anti‐human polysialic acid recombinant monoclonal antibody (Absolute Antibody) to the plasma reaction followed by goat‐anti‐mouse antibody conjugated to AF488 (Thermo Fisher Scientific) and incubated for 30 min. The sequential reactions were done at ambient temperature in the dark, then each tube was diluted 100‐fold with double filtered (0.1 μm cutoff) phosphate buffered saline and a 250 μL aliquot of the fully labeled sample was transferred into a 96 microwell U‐bottom plate well, in triplicate (Greiner Bio‐one, VWR).

### 2.6 Apogee A50 μFCM instrument settings and assay

The labeled EVs were measured using the Apogee A50 μFCM (Apogee Flow Systems) equipped with large angle light scatter (LALS) and small angle light scatter optical detectors. Light scatter was provided using the 488 nm laser (75 mW); Qdot® 565 signal was excited using the 488 nm laser (75 mW) with collected light passing a 545/70 nm bandpass filter; AF488 signal was generated using the 488 nm laser (50 mW) with collected light passing a 535/35 nm bandpass filter; and Cy5 signal was generated using the 638 nm laser (75 mW) with collected light passing a 680/35 nm bandpass filter. Every day prior to sample analysis, the cytometer was cleaned followed by running a silica‐polystyrene reference bead mix to verify instrument sensitivity and resolution (Apogee Mix Standards 1493, Apogee Flow Systems). Serial dilution of a subset of patient sample plasma was done to confirm analysis of single EVs, and signal events were triggered by light scatter only. Silica standards were used to assess the relative size range of the EVs. For each detector channel, the Gain was set at 1. Samples were run at a flow rate of 3.01 μL min^−1^ for up to 2 min or until 5,000,000 events were recorded, whichever came first, and over an 8‐h sampling window for the 96‐well plate. Conventional manual gating analysis of μFCM data was performed using Histogram version 255.0.0.80 software (Apogee Flow Systems). See Tables S1 and S2 for μFCM settings and MIFlowCyt/MISEV compliant items.^23^

### 2.7 Processing μFCM data

Patient μFCM FCS files were analyzed using a custom MATLAB (version R2017a) script (MathWorks). Within each file, signal intensities for all channels were log transformed and binned into 16‐groups per channel unless stated otherwise. Four different bivariate histograms of particle concentration were created: (1) LALS and PSMA stain intensity, (2) LALS and ghrelin probe stain intensity, (3) PSMA stain intensity and ghrelin probe stain intensity, and (4) LALS and polysialic acid stain intensity. Each bivariate histogram contained 256 regions of interest (ROIs, 16 × 16 bins). Particle concentration in each ROI was averaged over the three replicates per patient.

### 2.8 Descriptive statistical analysis

Unless stated otherwise, all statistics were calculated using Python 3.6.5 software using sklearn, scipy, and scikits‐bootstrap packages. When comparing 2 groups, Mann–Whitney *U*‐tests were used for interval data and Fisher's exact tests were used for binary categorical variables. ROC curves were compared by DeLong's method using the ‘pROC’ package in R software. When possible, ROC cutoff values were determined using at least 95% sensitivity and maximum specificity. Confidence intervals were determined with 100,000 resamples of bias‐corrected and accelerated bootstrapping.

### 2.9 Modeling statistics

The EVMAP machine learning model was created with the XGBoost algorithm using R version 3.4.1 software.^17^, ^24^ ROIs with minimal clinical value were removed via recursive feature elimination which used 10 repeats of 5‐fold cross‐validation (where all patients were divided into 5 different subgroups) using the caret package. Recursive feature elimination removed 10% (GG ≥2 and GG ≥3 cancers) or 25% (GG ≥1 cancer) of features in each iteration. The random forest model was used to determine the optimal features with the area under the curve (AUC) metric for ranking subsets of features. AUC values from all repeats of cross‐validation were averaged for each subset of features. After the optimal subset of ROIs were determined, machine learning models were created with optimal XGBoost model parameters determined by grid searching with 10 repeats of 5‐fold cross‐validation. The following XGBoost parameters were tested: *n*rounds (100, 150, 200, and 250), max_depth (3–5), and eta (0.01 and 0.1) using AUC for ranking parameter combinations.

Using the optimal XGBoost parameters, 500 different XGBoost models were created with 500 different randomly chosen subsets of patients which were developed by 5‐fold cross‐validation repeated 100 times. Patients in the training group (80% of the total cohort) were used for model creation and the model was evaluated on the held‐out group (the remaining 20% of patients). The test set was only used for evaluation, not for model development. Using only probabilities from the held‐out group, each patient had 100 different probabilities of GG ≥3 cancer which was averaged into one probability represented as the EVMAP predictive model. Then the EVMAP predictive model and six clinical features (age, ethnicity, family history of any prostate cancer, previous negative prostate biopsy, PSA levels, and DRE) were used as inputs for logistic regression models and evaluated using 5‐fold cross‐validation to generate the patients' EV‐Fingerprint Score.^17^ Prior to model training, missing clinical data was imputed with the median value from the training data.

## 3 RESULTS

Samples from 419 APCaRI participants which passed the eligibility and exclusion criteria were randomly pulled and analyzed with the μFCM assay. Four participants were removed due to missing PSA results. The remaining 415 patients were used for creating and evaluating machine learning models using 5‐fold cross‐validation. From the biopsies of the 415 patients; 157 (38%) had a negative biopsy, 258 (62%) were diagnosed with any prostate cancer and 73 (18%) were diagnosed with GG ≥3 cancer (Table 1). PSA, age, and diabetes incidence increased with increasing in cohorts with higher prostate cancer grade groups (Table 1).

**TABLE 1:** Patient characteristics, *n* = 415 cohort size.

	Negative biopsy	GG1	GG2	GG3–5
Patients, *n* (%)	157 (38%)	90 (22%)	95 (23%)	73 (18%)
PSA, ng/mL, median (IQR)	6.0 (3.8–7.7)	5.6 (4.4–8.4)	7.0 (5.3–9.7)	8.9 (6.8–17)
Age, years, median (IQR)	61 (56–67)	61 (56–68)	64 (58–69)	65 (58–71)
DRE, *n* (% abnormal)	31 (20%)	26 (29%)	28 (29%)	26 (36%)
Previous negative biopsy, *n* (%)	27 (17%)	5 (5.5%)	6 (6.3%)	7 (9.6%)
Race (Black), *n* (%)	4 (2.5%)	2 (2.2%)	1 (1.1%)	1 (1.4%)
Any family history of prostate cancer (first and second degree), *n* (%)	47 (30%)	30 (33%)	35 (37%)	25 (35%)
Body mass index, median (IQR)	27 (24–30)	26 (24–30)	28 (24–30)	28 (25–31)
Diabetes, *n* (%)	8 (5.7%)	5 (6.0%)	10 (11%)	11 (16%)

Abbreviations: GG, grade group prostate cancer; IQR, interquartile range.

Patient standard of care (SOC) clinical features were sorted according to GG score and listed in Tables 2–4. All clinical features were individually available in at least 83% of patients with PSA and age available for all patients. Men diagnosed with any prostate cancer versus no prostate cancer exhibited higher median age, higher PSA, and a higher likelihood of an abnormal DRE (Table 2).

**TABLE 2:** Predicting GG ≥1 prostate cancer using various clinical features, PCPTRC 2.0 representing six clinical features, EVMAP, and EV‐Fingerprint with the 415‐patient cohort.

	GG = 0	GG ≥1	*p* value	ROC AUC (CI)	Cutoff (≥)	Sensitivity, % (CI)	Specificity, % (CI)	PPV, % (CI)	NPV, % (CI)
Patients, *n*	157 (38%)	258 (62%)							
Race (Black), *n* (%)	4 (2.6%)	4 (1.6%)	0.49	0.51 (0.50–0.52)		98 (96–100)	2.6 (0.66–6.3)	62 (57–67)	50 (0.0–80)
Previous negative biopsy, *n* (%)	27 (17%)	18 (7.0%)	**0.0018**	0.55 (0.52–0.59)		93 (89–96)	17 (12–24)	65 (60–70)	60 (44–73)
Family history of prostate cancer, *n* (%)	47 (30%)	90 (35%)	0.33	0.53 (0.50–0.57)		35 (29–41)	70 (62–77)	66 (57–73)	40 (34–45)
Diabetes, *n* (%)	8 (5.7%)	26 (11%)	0.097	0.53 (0.50–0.55)		11 (7.3–15)	94 (89–97)	76 (60–89)	38 (33–43)
BMI, median (IQR)	27 (24–30)	27 (24–31)	0.41	0.52 (0.46–0.58)	>21.33	95 (91–97)	4.1 (1.6–8.9)	64 (59–69)	31 (11–58)
DRE, *n* (% abnormal)	31 (20%)	80 (31%)	**0.012**	0.56 (0.51–0.60)		31 (26–37)	80 (73–86)	72 (63–80)	42 (36–47)
Age, years, median (IQR)	61 (56–67)	63 (58–69)	**0.0031**	0.59 (0.53–0.64)	51.42	95 (91–97)	13 (8.3–19)	64 (59–69)	61 (42–76)
PSA, ng/mL, median (IQR)	6.0 (3.8–7.7)	7.0 (5.1–10)	**<0.0001**	0.63 (0.58–0.68)	3.400	95 (91–97)	18 (12–24)	65 (60–70)	67 (50–79)
PCPTRC 2.0, median (IQR)	72 (68–79)	68 (58–74)	**<0.0001**	0.69 (0.63–0.74)	82.00	96 (93–98)	15 (10–22)	65 (60–70)	71 (53–84)
EVMAP, median (IQR)	69 (52–80)	69 (56–84)	0.20	0.54 (0.50–0.59)	34.20	95 (91–97)	5.7 (2.7–10)	62 (57–67)	39 (20–61)
EVMAP + PSA + Age, median (IQR)	58 (48–66)	63 (54–73)	**<0.0001**	0.64 (0.58–0.69)	45.25	95 (91–97)	18 (12–25)	65 (60–70)	67 (50–79)
EV‐Fingerprint, median (IQR)	56 (45–65)	65 (55–78)	**<0.0001**	0.69 (0.63–0.74)	42.00	95 (91–97)	20 (15–27)	66 (61–71)	70 (55–82)

*Note*: All cutoffs were chosen for maximum specificity while maintaining at least 95% sensitivity. The bold values are *p*–values less than 0.05 and are considered statistically significant.

Abbreviations: BMI, body mass index; CI, confidence interval (95%); DRE, digital rectal exam; EVMAP, extracellular vesicle machine learning analysis platform; GG, grade group prostate cancer; NPV, negative predictive value; PCPTRC 2.0, six clinical features from the prostate cancer prevention trial risk calculator 2.0; PPV, positive predictive value; ROC AUC, receiver operating characteristic area under the curve.

EV‐Fingerprint showed good calibration compared to the prostate biopsy results, with the predicted probability of GG ≥3 cancer from the test closely matching the true risk as described by the biopsy results (Figure S1).

The accuracy of predicting prostate cancer with EV‐Fingerprint was compared to the online prostate cancer risk calculator PCPTRC 2.0 that uses age, ethnicity, family history of prostate cancer, previous negative prostate biopsy, PSA levels, and abnormal DRE data in the model. Tables 2–4 lists the discrimination capabilities for the following models: PCPTRC 2.0, EVMAP, EVMAP plus PSA levels and age analyzed with logistic regression (EVMAP + PSA + age), and EV‐Fingerprint, which is EVMAP plus the PCPTRC 2.0 high grade prostate cancer probability analyzed with logistic regression. The prediction of GG ≥1 cancer with EV‐Fingerprint showed a larger specificity over PCPTRC 2.0 (20% specificity for EV‐Fingerprint versus 15% specificity for PCPTRC 2.0; Table 2) along with an equal AUC and similar NPV (both at 0.69 AUC and 70%–71% NPV). The prediction of GG ≥2 cancer with EV‐Fingerprint showed higher AUC but lower specificity and NPV versus PCPTRC 2.0 only (Table 3). The prediction of GG ≥3 cancer with EV‐Fingerprint showed a superior and significant AUC, specificity and NPV over PCPTRC 2.0 (0.81 AUC, 41% specificity, 97% NPV versus 0.73 AUC, 24% specificity, 95% NPV; Table 4).

**TABLE 3:** Predicting GG ≥2 prostate cancer using various clinical features, PCPTRC 2.0 representing six clinical features, EVMAP, and EV‐Fingerprint with the 415‐patient cohort.

	GG ≤1	GG ≥2	*p* value	ROC AUC (CI)	Cutoff (≥)	Sensitivity % (CI)	Specificity % (CI)	PPV % (CI)	NPV % (CI)
Patients, *n*	247 (60%)	168 (40%)							
Race (Black), *n* (%)	6 (2.5%)	2 (1.2%)	0.48	0.51 (0.50–0.52)		99 (96–100)	2.5 (0.86–5.1)	41 (36–45)	75 (20–100)
Previous negative biopsy, *n* (%)	32 (13%)	13 (7.8%)	0.11	0.53 (0.50–0.55)		92 (87–96)	13 (9.2–18)	42 (37–47)	71 (56–83)
Family history of prostate cancer, *n* (%)	77 (31%)	60 (36%)	0.34	0.52 (0.50–0.57)		36 (29–44)	69 (63–74)	44 (35–52)	61 (56–67)
Diabetes, *n* (%)	13 (5.8%)	21 (13%)	**0.017**	0.54 (0.51–0.57)		13 (8.6–19)	94 (90–97)	62 (44–77)	61 (55–66)
BMI, median (IQR)	27 (24–30)	28 (25–31)	0.98	0.55 (0.49–0.61)	>21.23	95 (90–97)	3.6 (1.5–7.0)	42 (37–47)	47 (21–73)
DRE, *n* (% abnormal)	57 (23%)	54 (33%)	**0.042**	0.55 (0.51–0.59)		33 (26–40)	77 (71–82)	49 (39–58)	63 (57–68)
Age, years, median (IQR)	61 (56–67)	64 (58–71)	**0.00066**	0.61 (0.56–0.67)	52.40	95 (90–97)	15 (11–20)	43 (38–49)	80 (65–90)
PSA, ng/mL, median (IQR)	6.0 (4.1–7.8)	7.7 (5.6–12)	**<0.0001**	0.69 (0.64–0.74)	4.000	95 (90–97)	23 (18–29)	46 (40–51)	86 (76–93)
PCPTRC 2.0, median (IQR)	8.0 (5.0–12)	13 (8.0–20)	**<0.0001**	0.70 (0.65–0.75)	5.000	96 (92–98)	24 (19–30)	46 (41–52)	90 (80–95)
EVMAP, median (IQR)	37 (35–43)	41 (35–54)	**0.00021**	0.61 (0.55–0.66)	31.37	95 (90–97)	3.6 (1.7–6.6)	40 (35–45)	50 (23–71)
EVMAP + PSA + Age, median (IQR)	30 (22–41)	48 (33–70)	**<0.0001**	0.74 (0.69–0.79)	18.37	95 (90–97)	17 (12–22)	44 (39–49)	82 (69–91)
EV‐Fingerprint, median (IQR)	29 (23–40)	46 (32–71)	**<0.0001**	0.73 (0.68–0.78)	19.76	95 (90–97)	19 (14–24)	44 (39–49)	84 (71–92)

*Note*: All cutoffs were chosen for maximum specificity while maintaining at least 95% sensitivity. The bold values are *p*–values less than 0.05 and are considered statistically significant.

Abbreviations: BMI, body mass index; CI, confidence interval (95%); DRE, digital rectal exam; EVMAP, extracellular vesicle machine learning analysis platform; GG, grade group prostate cancer; NPV, negative predictive value; PCPTRC 2.0, six clinical features from the prostate cancer prevention trial risk calculator 2.0; PPV, positive predictive value; ROC AUC, receiver operating characteristic area under the curve.

**TABLE 4:** Predicting GG ≥3 prostate cancer using various clinical features, PCPTRC 2.0 representing six clinical features, EVMAP, and EV‐Fingerprint with the 415‐patient cohort.

	GG ≤2	GG ≥3	*p* value	ROC AUC (CI)	Cutoff (≥)	Sensitivity % (CI)	Specificity % (CI)	PPV % (CI)	NPV % (CI)
Patients, *n*	342 (82%)	73 (18%)							
Race (Black), *n* (%)	7 (2.1%)	1 (1.4%)	1.0	0.50 (0.50–0.51)		99 (92–100)	2.1 (0.89–4.1)	18 (14–22)	88 (33–100)
Previous negative biopsy, *n* (%)	38 (11%)	7 (9.6%)	0.84	0.51 (0.50–0.53)		90 (82–96)	11 (8.1–15)	18 (14–22)	84 (71–93)
Family history of prostate cancer, *n* (%)	112 (33%)	25 (35%)	0.78	0.51 (0.50–0.53)		35 (24–46)	67 (62–72)	18 (12–25)	83 (78–87)
Diabetes, *n* (%)	23 (7.3%)	11 (16%)	**0.034**	0.54 (0.50–0.59)		16 (8.5–27)	93 (89–95)	32 (18–50)	83 (79–87)
BMI, median (IQR)	27 (24–30)	28 (25–31)	0.56	0.55 (0.47–0.63)	21.13	95 (87–99)	3.2 (1.4–5.8)	18 (14–22)	75 (38–93)
DRE, *n* (% abnormal)	85 (25%)	26 (36%)	0.080	0.55 (0.50–0.61)		36 (25–47)	75 (70–79)	23 (16–32)	84 (80–88)
Age, years, median (CI)	62 (57–68)	65 (58–71)	**0.021**	0.60 (0.53–0.67)	53.35	96 (89–99)	14 (10–18)	19 (16–24)	94 (83–98)
PSA, ng/mL, median (CI)	6.2 (4.6–8.4)	8.9 (6.8–17)	**<0.0001**	0.73 (0.66–0.79)	4.200	95 (86–99)	21 (17–26)	20 (16–25)	95 (87–99)
PCPTRC 2.0, median (IQR)	9.0 (6.0–13)	15 (10–22)	**<0.0001**	0.73 (0.66–0.79)	6	95 (87–99)	24 (20–29)	21 (17–26)	95 (88–99)
EVMAP, median (IQR)	4.5 (2.2–12)	17 (5.9–41)	**<0.0001**	0.75 (0.69–0.81)	2.641	95 (87–99)	32 (28–38)	23 (18–28)	97 (91–99)
EVMAP + PSA + Age, median (IQR)	9.4 (6.7–14)	21 (12–61)	**<0.0001**	0.80 (0.74–0.85)	7.244	95 (87–99)	31 (26–36)	23 (18–27)	96 (91–99)
EV‐Fingerprint, median (IQR)	9.0 (6.5–14)	21 (13–68)	**<0.0001**	0.81 (0.75–0.86)	7.848	95 (87–99)	41 (36–46)	25 (21–31)	97 (93–99)

*Note*: All cutoffs were chosen for maximum specificity while maintaining at least 95% sensitivity. The bold values are *p*–values less than 0.05 and are considered statistically significant.

Abbreviations: BMI, body mass index; CI, confidence interval (95%); DRE, digital rectal exam; EVMAP, extracellular vesicle machine learning analysis platform; GG, grade group prostate cancer; NPV, negative predictive value; PCPTRC 2.0, six clinical features from the prostate cancer prevention trial risk calculator 2.0; PPV, positive predictive value; ROC AUC, receiver operating characteristic area under the curve.

The AUC of EVMAP for predicting GG ≥3 prostate cancer was significantly improved by adding PSA and age (0.75 vs. 0.80 AUC, respectively) and then further improved by including the PCPTRC 2.0 output (EV‐Fingerprint; 0.81 AUC; Table 5). Although diabetes status was significantly different between patients with and without GG ≥3 prostate cancer (7.3% vs. 16%, *p* = 0.034), adding this feature to the predictive model did not increase model AUC since it remained at 0.81. Using DRE as an additional feature with EVMAP, PSA, and age did not increase model AUC for predicting GG ≥1, GG ≥2, or GG ≥3 prostate cancers. When following the 157 patients with negative initial prostate biopsies for 5 years, five of these patients had subsequent prostate biopsies with GG 3 or greater prostate cancer (data not shown). All five patients had EV‐Fingerprint scores above the threshold and were flagged as high risk by the model.

**TABLE 5:** Comparison of AUC values for logistic regression models using EVMAP, various clinical features, PCPTRC 2.0, and EV‐Fingerprint.

	ROC AUCs for predicting prostate cancer		
Features	GG ≥1	GG ≥2	GG ≥3
EVMAP	0.54	0.61	0.75
PSA + Age	0.64	0.70	0.71
EVMAP + PSA + Age	0.64	0.74	0.80
*p* value	*0.85*	***0.027***	***0.0042***
PSA + Age + DRE	0.64	0.70	0.72
EVMAP + PSA + Age + DRE	0.63	0.74	0.80
*p* value	*0.77*	***0.037***	***0.03***
PCPTRC 2.0	0.69	0.70	0.73
EV‐Fingerprint	0.69	0.73	0.81
*p* value	*0.86*	*0.13*	***0.0053***

Abbreviations: DRE, digital rectal exam; EVMAP, extracellular vesicle machine learning analysis platform; GG, grade group prostate cancer; PCPTRC 2.0, prostate cancer prevention trial risk calculator 2.0; ROC AUC, receiver operating characteristic area under the curve.

The italic values are *p*–values, and the bold values are p–values less than 0.05 and are considered statistically significant.

Decision curve analysis showed that EV‐Fingerprint had a higher net benefit than PCPTRC alone at nearly all threshold probabilities commonly used in clinical care for GG ≥1, GG ≥2, and GG ≥3 prostate cancers (Figure S2A,C,E). The corresponding percentage of biopsies avoided over nearly all threshold probabilities for GG ≥1, GG ≥2 and GG ≥3 prostate cancers showed that EV‐Fingerprint avoided more biopsies per 100 patients than PCPTRC 2.0 alone (Figure S2B,D,F). Various EV‐Fingerprint clinical care cutoffs were used to calculate the number of biopsies that could have been avoided, and the percentage of GG ≥1, GG ≥2 and GG ≥3 prostate cancers that could have had a delayed diagnosis, by using the EV‐Fingerprint test (Table 6 and Table S3).

**TABLE 6:** EV‐Fingerprint cutoffs to calculate the number of biopsies that could have been avoided, and the percentage of GG ≥1, GG ≥2 and GG ≥3 prostate cancers that could have had a delayed diagnosis.

	Biopsies		GG ≥1 cancer		GG ≥2 cancer		GG ≥3 cancer	
EV‐Fingerprint cutoff (%)	Performed (%)	Avoided (%)	Found (%)	Missed (%)	Found (%)	Missed (%)	Found (%)	Missed (%)
0	415 (100)	0 (0)	258 (100)	0 (0)	168 (100)	0 (0)	73 (100)	0 (0)
≥5	384 (93)	31 (7)	248 (96)	10 (4)	164 (98)	4 (2)	73 (100)	0 (0)
≥7.5	294 (71)	121 (29)	203 (79)	55 (21)	143 (85)	25 (15)	69 (95)	4 (5)
≥7.847	271 (65)	144 (35)	190 (74)	68 (26)	139 (83)	29 (17)	69 (95)	4 (5)
≥10	200 (48)	215 (52)	143 (55)	115 (45)	106 (63)	62 (37)	61 (84)	12 (16)

## 4 DISCUSSION

This prospective study of EV‐Fingerprint resulted in two clinically significant findings: (1) improved predictive accuracy for diagnosis of GG ≥3 cancer than SOC, and (2) if a biopsy would be performed for an EV‐Fingerprint test score with *a* ≥ 7.85% probability of GG ≥3 cancer, then 35% of biopsies would have been avoided and diagnosis of GG ≥3 cancer would only have been missed in 5% of the men. Additionally, comparison of the sensitivity and specificity of the Beta test at different risk threshold cutoffs shows that using a cutoff of ≥5% no GG ≥3 cancers would have been missed (Table 6).

We developed the Apogee A50 μFCM assay to accurately measure prostate cancer biomarkers on EV populations within patients' plasma and combined these data with our purpose‐built machine learning model to predict clinically relevant EVs within the whole sample population. Effective EV‐based cancer diagnostic tests use biomarkers that are; (1) stable and detectable by μFCM, (2) cancer‐specific, (3) differentiate between cancer cell‐EVs versus non‐cancer cell‐EVs (diagnostic), and (4) differentiate between indolent and high grade prostate cancer (prognostic).^25^ The EV biomarkers used in the EV‐Fingerprint test were PSMA,^26^ ghrelin‐growth hormone secretagogue receptor (ghrelin),^27^ and polysialic acid.^28^ The PSMA transmembrane protein is expressed 100–1000 fold more in higher grade prostate cancers than in the benign prostate.^29^ The hormone ghrelin, whose expression is regulated by insulin in cancer cells, stimulates cell proliferation in prostate cancer cell lines.^30^ Cellular glycan structure modifications in cancer cells compared to healthy cells of the same tissue are considered a hallmark of cancer.^31^ Polysialic acid, a carbohydrate polymer expressed on the surface of cell adhesion molecules, modulates cell–cell and cell‐matrix adhesion, migration, invasion, and metastasis and is strongly associated with aggressive prostate cancer phenotype and poor clinical prognosis.^28^, ^32^ Therefore, quantifying the levels of these three biomarkers in patient blood samples provides the prostate cancer specific, diagnostic, and prognostic data required to accurately differentiate GG ≥3 cancer from GG ≥2 cancer.

The EV‐Fingerprint test outperformed PCPTRC 2.0 alone with a higher net benefit across all threshold probabilities and the largest and most statistically significant AUC (0.81) for predicting patients with GG ≥3 cancer. This was also reflected in the specificity (highest at 41%), PPV (highest at 25%) and NPV (highest at 97%) results for the EV‐Fingerprint models predicting GG ≥3 cancer. These values indicate that if the calculated test score of a patient with GG ≥3 cancer is low (i.e., below 7.85%), then the probability that the patient will not show GG ≥3 cancer on biopsy is very high (97%). Therefore, the EV‐Fingerprint test could improve on the current SOC of all patients undergoing prostate biopsy.

Features of the EV‐Fingerprint test in common with other prostate cancer diagnostic tests include: blood test with no DRE (4K Score, CNI 2nd Opinion, Iso PSA, Prostate Mitomic test and PHI), immunogenic detection of prostate cancer biomarkers (4K Score, IsoPSA, and PHI), and prostate cancer risk score calculated using an algorithm (4K Score, CNI 2nd Opinion, ExoDx Prostate, MyProstateScore, PHI, and SelectMDx).^33^, ^34^, ^35^, ^36^, ^37^, ^38^, ^39^, ^40^, ^41^, ^42^, ^43^, ^44^ Although head‐to‐head studies between EV‐Fingerprint and these other tests have not been done yet, the AUC for EV‐Fingerprint was similar or higher than these other biofluid tests (Table S4).^45^ Features unique to the EV‐Fingerprint test include (1) quantification of prostate cancer‐biomarkers on plasma‐derived EVs using μFCM, (2) analysis of the EV data using the purpose‐built XGBoost machine learning algorithm, and (3) calculation of the EV‐Fingerprint risk score via combination of μFCM analysis with clinical data to predict the risk of GG ≥3 prostate cancer and avoid unnecessary biopsy.

The main study strengths are as listed here. Inclusion of a large, contemporary cohort of men consented using APCaRI's comprehensive data collection. The biological samples and clinical data were stored in a secure biorepository and data registry. Uniform patient biopsy protocols and synoptic reporting consistent with ISUP 2005 criteria were used.^6^, ^19^

The main study limitations are listed below. The percentage of non‐Caucasian men in the cohort was low (13%). Although the cohort diversity is low, all men without prior diagnosis of prostate cancer were eligible for recruitment and only 3.3% of Albertan men identify as Black on annual Census. GG 2 prostate cancer is considered by many to be significant depending on volume of disease and patient age. MRI guided prostate biopsy data may affect the EV‐Fingerprint test results. Recent studies have shown mpMRI can improve diagnostic yield of prostate biopsy^46^; however, the use of mpMRI to detect prostate cancer was not widely available in Alberta during this study and therefore was not included. It is possible that some men were taking finasteride or dutasteride for benign prostate hyperplasia when the blood sample was taken. Recent clinical trials show that finasteride and dutasteride can lower patient PSA levels, and finasteride appears to improve the sensitivity and AUC for cancer detection by PSA.^47^ Another limitation is associated with the high prostate cancer detection rate in the screening population; 62% and 18% of the pre‐diagnosis cohort were diagnosed with GG ≥1 and GG ≥3 cancer after biopsy, respectively. However, this is comparable to the percentage of men from the general Albertan population (60%) diagnosed with prostate cancer after biopsy. Other health jurisdictions (USA, UK, Australia) report 20%–30% prostate cancer diagnosis after the initial biopsy,^48^ similar to PSA screening which correctly diagnoses prostate cancer for approximately 25%–30% of the men tested, leading to unnecessary biopsies.^48^ Conversely, in Alberta, the decision to biopsy is triggered by a highly abnormal DRE and either a rapidly rising PSA or a PSA doubling time of 2 years or less.^49^ This results in fewer unnecessary biopsies and higher percentages of GG ≥1 and GG ≥3 cancer after biopsy.

## 5 CONCLUSIONS

This prospective study suggests that EV‐Fingerprint can accurately and with high specificity predict patients with GG ≥3 prostate cancer, and with very few missed GG ≥3 cancer cases. We are now conducting a larger prospective study for clinical validation of our next generation predictive prostate cancer test, with approximately 7‐fold more men consented from urology centers in Canada and the USA and with more ethnic diversity.

## AUTHOR CONTRIBUTIONS

**Adrian Fairey:** Conceptualization (equal); formal analysis (equal); investigation (equal); writing – original draft (equal). **Robert J. Paproski:** Conceptualization (equal); data curation (equal); formal analysis (equal); investigation (equal); methodology (equal); software (lead); validation (equal); visualization (equal); writing – original draft (equal); writing – review and editing (equal). **Desmond Pink:** Conceptualization (equal); data curation (equal); formal analysis (equal); investigation (equal); methodology (equal); supervision (equal); validation (equal); visualization (equal); writing – review and editing (equal). **Deborah L. Sosnowski:** Data curation (equal); investigation (equal); methodology (equal); validation (equal); visualization (equal). **Catalina Vasquez:** Formal analysis (equal); investigation (equal); project administration (equal); resources (equal); writing – review and editing (equal). **Bryan Donnelly:** Formal analysis (equal); investigation (supporting); writing – review and editing (supporting). **Eric Hyndman:** Formal analysis (equal); investigation (equal); writing – review and editing (supporting). **Armen Aprikian:** Formal analysis (equal); investigation (equal); writing – review and editing (supporting). **Adam Kinnaird:** Formal analysis (equal); investigation (equal); writing – review and editing (equal). **Perrin H. Beatty:** Writing – original draft (equal); writing – review and editing (equal). **John D. Lewis:** Conceptualization (lead); data curation (equal); formal analysis (equal); funding acquisition (lead); investigation (equal); project administration (lead); resources (lead); supervision (equal); validation (equal); visualization (equal).

## FUNDING INFORMATION

Alberta Innovates – Health Solutions (AIHS 201201259). Bird Dogs – Alberta Cancer Foundation: Prostate Cancer Research Plan (ACF 26001). Prostate Cancer Canada (PCC MTA TAG2014‐03). JDL holds the Bird Dogs Chair in Translational Oncology funded by the Alberta Cancer Foundation.

## CONFLICT OF INTEREST STATEMENT

RJP, DP, CV, and JDL are employees and shareholders in Nanostics Inc. AF, EH, and PHB are employees of Nanostics Inc. AA is a board member of Nanostics Inc. BD, AK and DLS have no conflicts of interest to disclose.

## ETHICS APPROVAL

The clinical study was approved by the Health Research Ethics Board of Alberta under the APCaRI‐01 protocol (HREBA‐CC‐18‐0513).

## Supporting information


Figure S1.
Figure S2.
Table S1.
Table S2.
Table S3.
Table S4.

## Data Availability

The data generated in this study are available within the article and its supplementary material.
