# Supplementary material for: Clinical analysis of EV‐Fingerprint to predict grade group 3 and above prostate cancer and avoid prostate biopsy
Source: Cancer Med. 2023 Jun 17;12(15):15797–808. doi: 10.1002/cam4.6216 (PMC10469644; doi:10.1002/cam4.6216)
Supplement: Supplementary file 1 — Figure S1. Figure S2. Table S1. Table S2. Table S3. Table S4. [file CAM4-12-15797-s001.docx]

**Supplementary Material**

**Clinical analysis of EV-Fingerprint to predict Grade Group 3 and Above Prostate Cancer and Avoid Prostate Biopsy**

Adrian Fairey^1,2^, Robert J Paproski^2,3^, Desmond Pink^2,3^, Deborah L. Sosnowski^3^, Catalina Vasquez^2,3^, Bryan Donnelly^4^, Eric Hyndman^3,4^, Armen Aprikian^3,5,^ Adam Kinnaird^1^, Perrin H. Beatty^2,3^, and John D. Lewis^2,3^*

^1^Kipnes Urology Centre, Kaye Edmonton Clinic, 11400 University Ave, Edmonton, AB, T6G 1Z1.

^2^Nanostics Inc., 4550 10230 Jasper Ave, Edmonton, AB, Canada, T5J 4P6.

^3^Department of Oncology, Katz Group Centre, 5-142 114 St and 87 Avenue University of Alberta, Edmonton, AB, T6G 2E1.

^4^Prostate Cancer Centre, University of Calgary, 7007 14 St SW Suite 6500, Calgary, AB T2V 1P9.

^5^Department of Surgery, McGill University, Montreal General Hospital, 1650 Cedar Avenue, Montreal, QC H3G 1A4

Corresponding Author:

*John D. Lewis, Department of Oncology, 5-142C Katz Group Building, University of Alberta, Edmonton, AB T6G 2E1 Canada

Email: [jdlewis@ualberta.ca](mailto:jdlewis@ualberta.ca)

Running Title: EV-Fingerprint predicts GG ≥ 3 prostate cancer.

Keywords: Biomarkers, Clinical Cancer Research, Prostate cancer, Screening.

Figures S1-S2

Tables S1-S4


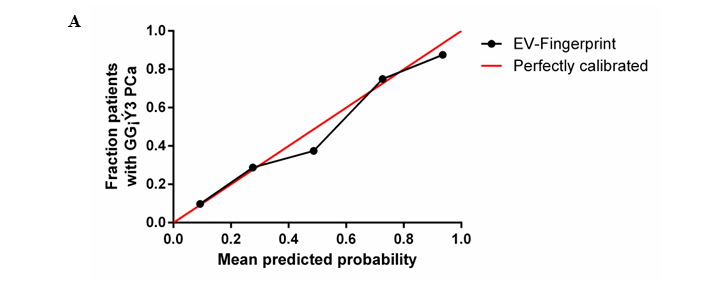


Figure S1. Calibration curve of EV-Fingerprint scores showing actual and predicted GG ≥ 3 prostate cancer.


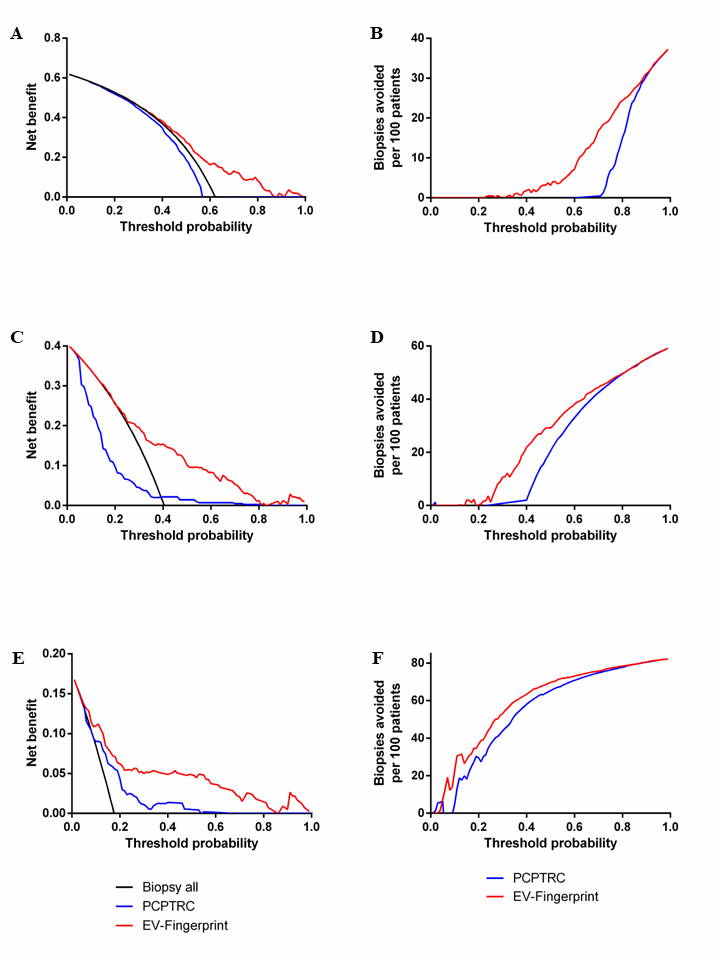


Figure S2. The decision curve analysis (DCA) net benefit and biopsies avoided per 100 patients analyzed for a 415-patient cohort with the assumptions that a biopsy should be performed if the patient has: A and B) any form of prostate cancer, or C and D) GG ≤ 2 prostate cancer, or E and F) GG ≥ 3 prostate cancer.

Table S1. Microflow cytometry settings.

| **Platform** | Apogee A50 MP | S/N 0027 |  |  |
| --- | --- | --- | --- | --- |
|  |  |  |  |  |
| **Parameter** | **Setting** |  |  |  |
| Sample Flow Rate | 3.01 µL/min |  |  |  |
| Pressure | 150 units |  |  |  |
| Acquisition time | 60 sec |  |  |  |
| Sample Dilution | 100x with PBS |  |  |  |
| Volume/well | 250 µL |  |  |  |
| Sample volume | 10 μL |  |  |  |
| Diluent volume | 990- Ab vol μL |  |  |  |
| Event Trigger | LALs unless stated otherwise | |  |  |
|  |  |  |  |  |
| **Channel** | **Laser Power (mW)** | **PMT** | **Gain** | **Threshold** |
| 405nm | 75 |  |  |  |
| 488nm | 50 |  |  |  |
| 561nm | N/A |  |  |  |
| 638nm | 75 |  |  |  |
| 405-SALS |  | 342 | 1.0 | 20 |
| 405-LALS |  | 350 | 1.0 | 48 |
| 405-Red |  | 350 | 1.0 |  |
| 405-Green |  | 500 | 1.0 |  |
| 488-Green |  | 450 | 1.0 |  |
| 488-Orange |  | 550 | 1.0 |  |
| 488-Red |  | 580 | 1.0 |  |
| 561-Orange |  | NA | 1.0 |  |
| 561-Red |  | NA | 1.0 |  |
| 638-Red |  | 550 | 1.0 |  |
| 638-Far Red |  | NA | 1.0 |  |
|  |  |  |  |  |
| **Beads** | **Product number** | **Lot number** | **Expiration** |  |
| Monitoring | Apogee 1493 | CAL0093  CAL0095 | 09/08 2022  13/02/2023 |  |

Table S2. MIFlowCyt / MISEV Compliant Items for the standardized reporting of extracellular vesicle flow cytometry experiments ^51^.

| **Requirement** | **Please Include Requested Information** |
| --- | --- |
| 1.1. Purpose | To assess the use of liquid biopsy (platelet depleted plasma) as a source of extracellular vesicles for detection of different prostate cancer biomarkers. |
| 1.2. Keywords | Prostate cancer, plasma, biomarker, extracellular vesicle |
| 1.3. Experiment variables | Patient plasma, risk of cancer. Specific details in Methods. |
| 1.4. Organization name and address | John D. Lewis Lab, University of Alberta, Dept of Experimental Oncology, 5142 Katz Group Centre Edmonton, Alberta, Canada T6G 2E1 |
| 1.5. Primary contact name and email address | John D. Lewis, jdlewis@ualberta.ca |
| 1.6. Date or time period of the experiment | 2017 - 2020 |
| 1.7. Conclusions | Our data demonstrate that extracellular vesicles, in this primary study may be useful for the detection of prostate cancer and in conjunction with machine learning algorithms may be predictive or aggressive cancer risk. |
| 1.8. Quality control measures | 1493 Apogee Bead Mix, daily instrument monitoring. |
| 2.1.1.1. (2.1.2.1., 2.1.3.1.) Sample description | Platelet-free plasma from patients at risk for prostate cancer. |
| 2.1.1.2. Biological sample source description | Pre-biopsy plasma samples from 215 men suspected of prostate cancer were acquired from the Alberta Prostate Cancer Research Initiative (APCaRI) biorepository(35). The clinical study was approved by the Health Research Ethics Board of Alberta under the APCaRI-01 protocol (HREBA-CC-18-0513). |
| 2.1.1.3. Biological sample source organism description | Human, EDTA plasma |
| 2.1.2.2. Environmental sample location | NA |
| 2.3. Sample treatment description | Human plasma samples   1. Frozen plasma samples were thawed, centrifuged at 16,000xg for 30 minutes to remove large debris and platelet particles, and incubated with five µg/mL mouse anti-PSMA (J591) antibody and a 1:50 dilution of secondary Qdot565-conjugated donkey anti-mouse IgG antibody. 2. Samples were also incubated with 0.133 mM Cy5.5-ghrelin probe containing the first 18 amino acids of ghrelin. 3. Thirty minutes after probe incubation, samples were diluted 100-fold in double-filtered (0.1 µm) phosphate-buffered saline and analyzed with the Apogee A50 microflow cytometer using a flow rate of 3.01 µL/minute. Samples were run for up to 2 minutes or until 5,000,000 events were recorded, whichever came first. 4. Plasma from each patient was run in triplicate. Conventional manual gating analysis of µFCM data was performed using Histogram version 255.0.0.80 software (Apogee Flow Systems). |
| 2.4. Fluorescence reagent(s) description | 1. Secondary Qdot565-conjugated donkey anti-mouse IgG antibody. 2. 0.133 mM Cy5.5-ghrelin probe |
| 3.1. Instrument manufacturers, model | 1. Apogee A50 MicroPlus Microflow cytometer (S/N 0027): Apogee Flow Systems |
| 3.3. Instrument configuration and settings | See Tables |
| 4.1. Compensation description | No compensation |
| 4.2. Data transformation details | NA |
| 4.3.1. Gate description | Defined by unstained controls. |
| 4.3.2. Gate statistics | Data provided as concentration (events/uL) |
| 4.3.3. Gate boundaries | Defined by unstained controls as well as autogating defined by ML algorithms |

**Table S3**. EV-Fingerprint cutoffs to calculate the number of biopsies that could have been avoided, and the percentage of GG ≥ 1, GG ≥ 2, and GG ≥ 3 prostate cancers that could have had a delayed diagnosis.

|  | **Biopsies** | | **GG ≥ 1 PCa** | | **GG ≥ 2 PCa** | | **GG3** ≥ **PCa** | |
| --- | --- | --- | --- | --- | --- | --- | --- | --- |
|  | **Performed (%)** | **Avoided**  **(%)** | **Found**  **(%)** | **Missed**  **(%)** | **Found**  **(%)** | **Missed (%)** | **Found (%)** | **Missed (%)** |
| **Biopsy all** | 415 (100%) | 0 (0%) | 415 (100%) | 0 (0%) | 415 (100%) | 0 (0%) | 415 (100%) | 0 (0%) |
| **Models/thresholds optimized for GG** ≥ **2 PCa** | | | | | | | | |
| **PCPTRC** | 348 (84%) | 67 (16%) | 235 (91%) | 23 (9%) | 161 (96%) | 7 (4%) | 71 (97%) | 2 (3%) |
| **EVMAP** | 397 (96%) | 18 (4%) | 244 (95%) | 14 (5%) | 159 (95%) | 9 (5%) | 70 (96%) | 3 (4%) |
| **EV-Fingerprint** | 366 (88%) | 49 (12%) | 238 (92%) | 20 (8%) | 159 (95%) | 9 (5%) | 72 (99%) | 1 (%) |
| **Models/thresholds optimized for GG** ≥ **3 PCa** | | | | | | | | |
| **PCPTRC** | 328 (79%) | 87 (21%) | 222 (86%) | 36 (14%) | 155 (92%) | 13 (8%) | 69 (95%) | 4 (5%) |
| **EVMAP** | 300 (72%) | 115 (28%) | 196 (76%) | 62 (24%) | 139 (83%) | 29 (17%) | 69 (95%) | 4 (5%) |
| **EV-Fingerprint** | 271 (65%) | 144 (35%) | 190 (74%) | 68 (26%) | 139 (83%) | 29 (17%) | 69 (95%) | 4 (5%) |

**Table S4.** Comparison of AUC values predicting high-grade prostate cancer between

EV-Fingerprint, PSA, and similar biofluid tests.

| **Features** | **Year and Reference** | **ROC AUCs** |
| --- | --- | --- |
| **EV-Fingerprint** | This study | 0.81 |
| **PSA** | This study | 0.66 |
| **4K Score** | 2008 ^44^ | 0.83 |
|  | 2015 ^41^ | 0.82 |
|  | 2016 ^45^ | 0.78 |
| **Prostate Health Index** | 2013 ^35^ | 0.73 |
|  | 2018 ^46^ | 0.76 |
| **Progensa PCA3** | 2013 ^35^ | 0.73 |
| **SelectMDx** | 2016 ^38^ | 0.76 |

Abbreviations: ROC AUC, receiver operating characteristic area under the curve.

PCPTRC 2.0, prostate cancer prevention trial risk calculator 2.0.
